# Norovirus Gastroenteritis among Hospitalized Patients, Germany, 2007–2012

**DOI:** 10.3201/eid2411.170820

**Published:** 2018-11

**Authors:** Frank Kowalzik, Harald Binder, Daniela Zöller, Margarita Riera-Montes, Ralf Clemens, Thomas Verstraeten, Fred Zepp

**Affiliations:** University Medical Centre of the Johannes Gutenberg University, Mainz, Germany (F. Kowalzik, H. Binder, D. Zöller, F. Zepp);; P95 Pharmacovigilance and Epidemiology Services, Leuven, Belgium (M. Riera-Montes, T. Verstraeten);; Takeda Pharmaceutical International AG, Zurich, Switzerland (R. Clemens)

**Keywords:** norovirus, gastroenteritis, disease burden, cost, hospitalization, nosocomial, viruses, Germany, enteric infections

## Abstract

We estimated numbers of hospitalizations for norovirus gastroenteritis (NGE) and associated medical costs in Germany, where norovirus testing is high because reimbursement is affected. We extracted aggregate data for patients hospitalized with a primary or secondary code from the International Classification of Diseases, 10th Revision (ICD-10), NGE diagnosis during 2007–2012 from the German Federal Statistics Office. We assessed reliability of the coding system in patient records from a large academic hospital. Approximately 53,000–90,000 NGE hospitalizations occurred annually in Germany (21,000–33,000 with primary and 32,000–57,000 with secondary ICD-10–coded NGE diagnoses). Rates of hospitalization with NGE as primary diagnosis were highest in children <2 years of age; rates of hospitalization with NGE as secondary diagnosis were highest in adults >85 years of age. The average annual reimbursed direct medical cost of NGE hospitalizations was €31–43 million. Among patients with a NGE ICD-10 code, 87.6% had positive norovirus laboratory results.

Norovirus is a leading cause of acute gastroenteritis (AGE) in all age groups and in some industrialized countries has overtaken rotavirus as the most frequent cause of AGE cases requiring hospitalization among children since rotavirus vaccination began (*1*–*5*). Because of its particularly high contagiousness (*6*), norovirus-related disease is a frequent cause of community and nosocomial AGE outbreaks. Hospital outbreaks of norovirus gastroenteritis (NGE) can lead to disruption of patient care and substantial economic costs associated with containment and ward closure (*7*–*9*).

In Germany, rotavirus and norovirus infections are notifiable to the Robert Koch Institute (RKI) (*10*). Rotavirus vaccine has been incrementally included in immunization schedules in 5 of the 16 federal states in Germany since 2008. In 2012, vaccination coverage rates ranged from 11% to 77%, and estimated national coverage was 32% (*11*). Universal immunization for infants was recommended in 2013 (*12*). On the basis of its own analyses of notifications, RKI has identified noroviruses as the most frequent cause of AGE; estimated annual incidence of NGE is 123–130 cases/100,000 population (*13*,*14*). The proportion of NGE notifications associated with hospitalized case-patients ranges from 8% to 26% annually (*13*,*14*).

With norovirus vaccines in development (*15*,*16*), baseline data on the NGE burden will be useful for informing decisions on potential future vaccine introduction and impact evaluation. A unique situation exists in Germany because NGE diagnosis is directly linked to reimbursement of hospital costs, providing an incentive for testing AGE cases for their etiology. The reimbursement for an AGE case requiring hospitalization increases by ≈€400 if the cause can be shown to be norovirus, rotavirus, or adenovirus.

The German Federal Statistics Office (DESTATIS) database records statistical information throughout Germany and has been used to conduct research across healthcare disciplines (*17*,*18*). We retrospectively queried the DESTATIS database and conducted a medical record review to estimate the age-stratified NGE burden among hospitalized AGE patients in Germany. We further describe some epidemiologic features and costs associated with NGE cases requiring hospitalization.

## Methods

The main study objective was to determine the number of NGE hospitalizations overall and stratified by age (<1, 1–17, 18–44, 45–64, 65–84 and >85 years) in Germany by using hospital discharge data obtained from DESTATIS. German inpatient data are recorded centrally and provided to DESTATIS. All hospitals are required by law to report patient information (for reimbursement purposes) on age, sex, duration of hospital stay, reason for admission, and discharge based on codes from the International Classification of Diseases, 10th Revision (ICD-10), and the Diagnosis-Related Group (DRG). We requested information on all hospitalizations resulting from AGE (ICD-10 codes A00–A09, representing all infectious intestinal diseases) for the period 2007–2012. In addition, we specifically requested information on hospitalizations with ICD-10 code A08.1 (NGE) as the primary or secondary diagnosis and a DRG code of G67 (esophagitis, gastroenteritis, gastrointestinal bleeding, ulcers, and miscellaneous digestive system disorders). Including the base DRG code G67 enabled us to differentiate between the different DRG codes in the same base DRG group (i.e., G67 A–D), which are linked to different reimbursement amounts. For comparison, we requested similar information on rotavirus hospitalizations (ICD-10 code A08.0). We also requested data on deaths related to the same codes as the primary diagnosis.

We assessed the direct hospitalization costs associated with NGE hospitalizations in Germany (primary diagnosis), which were available for the years 2007–2009. The DRG reimbursement data from DESTATIS are independent of the duration of hospital stay and do not include surcharges based on long-stay DRG or discounts based on short-stay DRG.

To validate the reporting system for NGE cases, we reviewed the clinical files of a random selection of 1,214 patients at the University Medical Centre Mainz (Mainz, Germany) who had been tested for norovirus. Demographic, clinical, and microbiologic information was extracted by using a standardized case report form.

The study protocol was reviewed by the local ethics committee in Mainz (Landesärztekammer Rheinland-Pfalz). Because this study was noninterventional and all data were anonymized and aggregated, a waiver for ethics approval was obtained.

### Statistical Analysis

We calculated incidence rates of hospitalizations with NGE by using annual population projections in Germany for the study period from DESTATIS GENESIS-Online (*19*). We calculated the proportions of all hospitalizations and all AGE hospitalizations that were attributable to NGE overall, by age group, and by study year.

We evaluated the total number of cases with NGE as a primary diagnosis per month of admission (available from 2009 onward) and by federal state (available for all study years) to reflect seasonal and regional distribution. To maintain confidentiality, DESTATIS applies a procedure whereby data from age groups that have <3 patients with the diagnostic condition per month are removed from the dataset. A maximum of 49 patients in any 1 year were deleted from the dataset as the result of the DESTATIS procedure, which is not expected to have affected the study findings.

The performance of the reporting system is expressed as a proportion of NGE-coded patients for whom we could identify a positive norovirus test result and the proportion of test-positive patients to whom an NGE code had been assigned; 95% CIs are provided. We performed the analyses by using SAS version 9.4 (SAS Institute Inc., Cary, NC, USA).

## Results

We identified a total of 408,124 hospitalizations with an NGE diagnosis in Germany during 2007–2012, for an annual average of 68,187 hospitalizations (Table 1). The overall number of NGE hospitalizations were consistently higher than the number of rotavirus hospitalizations for all age groups except children <2 years of age. Furthermore, for each of the study years, more deaths among hospitalized patients with an NGE diagnosis occurred than among those with rotavirus AGE (Table 1).

**Table 1:** Total annual number of AGE hospitalizations and deaths in hospital (all ages), Germany 2007–2012*

Pathogen	2007	2008	2009	2010	2011	2012	Average
Hospitalizations†							
Norovirus	53,701	67,130	63,307	91,001	67,934	66,051	68,187
Rotavirus	37,374	34,978	43,273	35,986	35,365	32,639	34,857
All causes	328,033	341,352	309,351	394,753	371,851	359,933	350,879
Deaths‡							
Norovirus	194	201	173	265	191	194	203
Rotavirus	30	55	56	53	42	40	46
All causes	2,046	2,532	2,510	2,981	3,262	3,420	2,792

*AGE, acute gastroenteritis. †All AGE (primary and secondary diagnoses). ‡AGE as primary diagnosis only.

### Hospitalizations with NGE as Primary Diagnosis

The annual number of hospitalizations with NGE as the primary diagnosis ranged from 21,442 to 33,440 (average 27,910) (Technical Appendix Table 1). NGE hospitalizations represented 11.5%–15.6% of all hospitalizations with AGE as the primary diagnosis annually. The overall incidence of hospitalization with NGE as the primary diagnosis in Germany during the study period was 3.4/10,000 population (range 2.6–4.1/10,000 population) (Technical Appendix Table 1).

**Figure 1:**
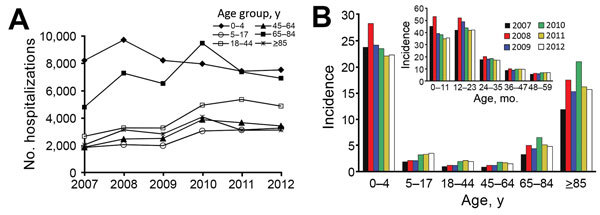
Annual number and incidence of hospitalizations for norovirus gastroenteritis as primary diagnosis, Germany, 2007–2012. A) Annual cases by age group; B) annual incidence (no. cases/10,000 population) by age group. Inset shows incidence by age group among children <5 years of age.

Among children <1 year of age, annual incidence ranged from 34.8 to 53.1/10,000 population, and among children 1 to <2 years of age, the annual incidence ranged from 41.8 to 52.0/10,000 population (Figure 1). After the age of 2 years, NGE hospitalization incidence rates decreased rapidly with age but increased again for persons >65 years of age, reaching 11.8 to 20.9/10,000 population in persons >85 years of age (Figure 1).

In each study year, the absolute number of NGE hospitalizations was highest among adults 65–84 years of age (range 4,800–9,495/y) and children <5 years of age (range 7,438–9,729/y) (Figure 1). In each study year, the overall rate of NGE hospitalizations was higher in females than males (rate ratio 1.2 for all study years). Among hospitalized patients with NGE, 194–265 deaths (average 203) occurred annually (Table 1), most (96.3%) of which occurred among adults >65 years of age (data not shown). The number of deaths in children <5 years of age ranged from 0 to 5 annually. 

### Hospitalizations with NGE as Secondary Diagnosis

**Figure 2:**
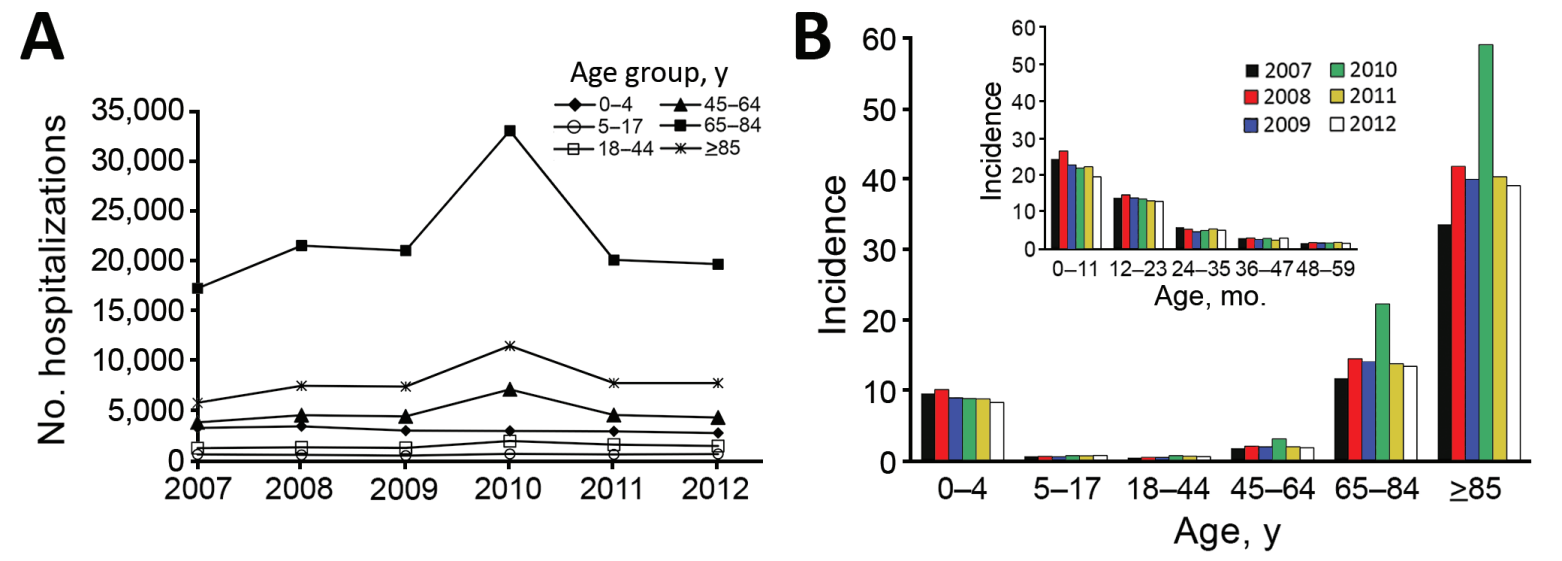
Annual number and incidence of hospitalizations for norovirus gastroenteritis as secondary diagnosis, Germany, 2007–2012. A) Annual cases by age group; B) annual incidence (no. cases/10,000 population) by age group. Inset shows incidence by age group among children <5 years of age.

During 2007–2012, an annual average of 40,278 (range 32,259–57,561) hospitalizations occurred with an NGE ICD-10 code A08.1 listed as a secondary diagnosis (Figure 2, panel A). In any study year, the number of hospitalizations with NGE as secondary diagnosis was 1.3–1.7 times higher than for those with NGE as a primary diagnosis. In contrast to NGE as a primary diagnosis, in which the highest rates were in children, the rate of hospitalizations with NGE as a secondary diagnosis was highest in adults >85 years of age (range 33.6–59.1/10,000 population) (Figure 2, panel B).

### Seasonal and Regional Distribution of NGE Hospitalizations (Primary Diagnosis)

**Figure 3:**
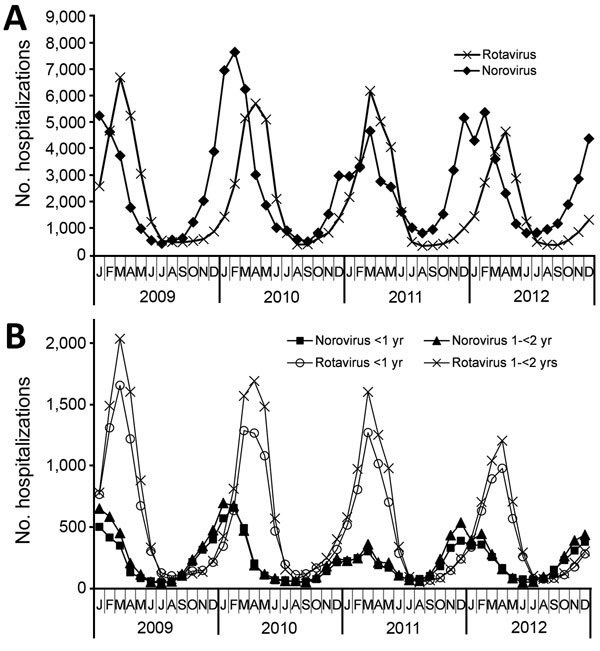
Monthly number of hospitalizations for rotavirus and norovirus gastroenteritis as primary diagnosis among all age groups (A) and among children 1 to <2 years of age (B), Germany, 2009–2012.

**Figure 4:**
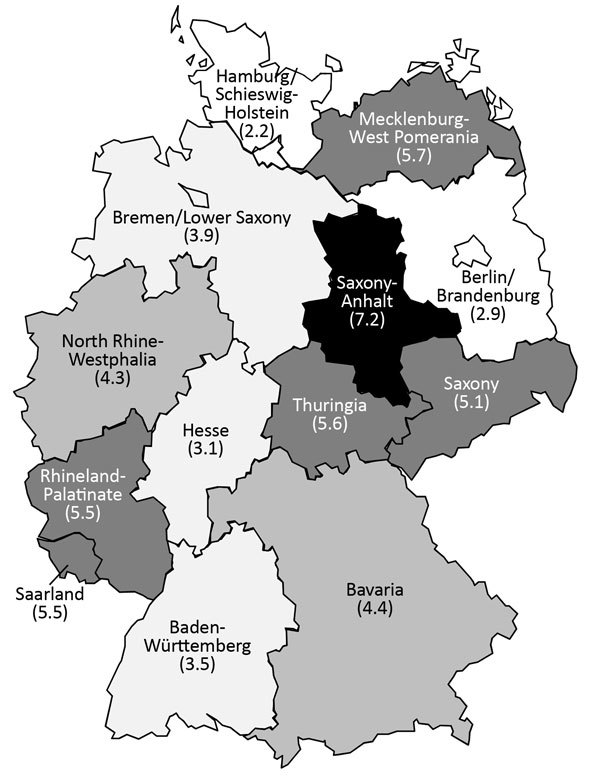
Regional distribution of norovirus hospitalizations among all age groups, by federal state, Germany, 2010. Numbers in parentheses indicate no. cases/10,000 population. Map template obtained from http://www.presentationmagazine.com/editable-maps/page/3.

NGE hospitalizations showed strong seasonality; the highest case numbers occurred during December–March for each of the study years (Figure 3). In 4 out of 5 study years, hospitalizations with a NGE diagnosis peaked before rotavirus hospitalizations. Regional distribution of NGE hospitalizations across Germany showed marked differences: >3-fold variations in the calculated region-specific incidence rates. In 2010, the year with the most NGE hospitalizations, the incidence of NGE hospitalizations was as low as 2.2/10,000 population in Hamburg/Schleswig-Holstein and as high as 7.2/10,000 population in Saxony-Anhalt. However, we found no consistent geographic pattern (Figure 4).

### Length of Stay and Cost for NGE Hospitalizations

We calculated 108,093–156,538 hospital bed days (average 131,411) each year for hospitalization with NGE as a primary diagnosis. The mean length of stay was 4.7 days (range 4.2–5.2 days); longer stays occurred among young children and older adults. Specifically, we identified hospital stays of 3.6–4.4 days in children <1 year of age, 3.0–3.7 days in children 1 to <2 years of age, 6.0–7.5 days in persons 65–84 years of age, and 7.3–8.9 days in persons >85 years of age. Across all age groups, the average duration of hospitalization was 3.4–4.5 times longer (17–18 days vs. 4–5 days) when NGE was a secondary diagnosis (Technical Appendix Table 2). The data did not enable us to determine the relative contribution of the NGE to the total duration of the hospitalization.

The fixed reimbursement per hospitalization with NGE as a primary diagnosis was €1,514 in 2007, €1,639 in 2008, and €1,692 in 2009. These amounts correspond to a total reimbursed cost to the German health system ranging from €31 to €42 million annually.

### Validation of the Base DRG Code G67 and ICD-10 A08.1

Among the 1,214 patients tested for norovirus for whom we reviewed the clinical files, 113 had positive and 1,101 negative results (Table 2). Of the 113 with positive results, 99 had an NGE ICD-10 code assigned, suggesting that 87.6% (95% CI 81.5%–93.7%) of the positive norovirus cases are captured by the coding system. Conversely, of the 104 patients with an NGE ICD-10 code, 99 had a positive norovirus result, suggesting that 95.2% (95% CI 91.1%–99.3%) of the coded NGE cases represent laboratory-confirmed norovirus cases.

**Table 2:** Concordance between ELISA norovirus tests and the disease-specific ICD-10 code for norovirus gastroenteritis as primary diagnosis, University Medical Centre of Mainz, Germany, 2011–2012*

Result	ICD-10 A08.1 code, no. (%)		
	No	Yes	Total
Norovirus negative	1,096	5	1,101 (90.7)
Norovirus positive	14	99	113 (9.3)
Total	1,110 (91.4)	104 (8.6)	1,214 (100)

*ICD10, International Classification of Diseases, 10th Revision.

## Discussion

We used national hospitalization statistics to estimate the overall burden of NGE hospitalizations in Germany. During 2007–2012, a total of 408,124 NGE-related hospitalizations occurred. On average, >60,000 hospitalizations occurred annually, including 27,910 hospitalizations with NGE as a primary diagnosis and 40,278 hospitalizations with NGE as a secondary diagnosis, as well as up to 265 NGE-related deaths at a hospital per year.

As reported in other studies of NGE epidemiology (*20*,*21*), the incidence of NGE hospitalizations by age follows a U-shaped curve, representing the highest rates in young children and the elderly. Among children <5 years of age, an annual average of >8,000 hospitalizations occurred with NGE as a primary diagnosis; among adults >65 years of age, the annual average was >10,000 hospitalizations.

We described hospitalizations with NGE as a primary or secondary diagnosis separately because we believe they represent different aspects of the burden caused by norovirus infections. The hospitalizations with NGE as a primary diagnosis are most likely representative of community-acquired infections, whereas the secondary NGE diagnoses are more likely to represent nosocomial infections. This assumption about secondary diagnoses might be wrong if patients entered the hospital with NGE occurring simultaneously with another disorder, where the NGE was judged to be a secondary cause of the hospitalization. This scenario might be especially true for older patients with co-occurring conditions that might have been exacerbated as a result of AGE. Also, several patients might have been hospitalized for reasons other than AGE but had onset of signs of NGE in the first 48 hours of hospitalization, which would indicate the patient acquired the infection before hospitalization.

Our estimate of the rate of hospitalization with NGE as a primary diagnosis is higher than the rate for community-acquired NGE estimated by Spackova et al. (*14*), based on German NGE reports made to RKI. That study estimated that 8%–19% of the 856,539 total NGE hospitalization cases over an 8-year period were community-acquired, corresponding to ≈8,500–20,000 community-acquired NGE hospitalization cases annually. The German Health Insurance Medical Service (Medizinischer Dienst der Krankenkassen [MDK]) (*22*) carries out a stringent quality-control program of DRG and ICD-10 codes reported by hospitals, as required by law. Because our results are based on data abstracted from this rigorously controlled reimbursement system, we believe our results are more likely to represent the actual burden of all NGE hospitalizations in Germany. Moreover, our validation of the test results in a subset of patients with AGE found a high reliability for the NGE ICD-10 code compared with actual testing, suggesting little overattribution of NGE in this setting. This finding is further supported by data provided in a study by Bernard et al*.* (*13*), which estimated norovirus cases to be underreported to RKI by a minimal underreporting factor of 1.7. Our estimates of the NGE hospitalization rate are also higher than previously reported estimates from countries such as the Netherlands (*23*), the United States (*24*), and Canada (*25*) but are comparable to a recent study in England (*26**,**27*); (Table 3). Our estimates of annual NGE hospitalizations resulting in death (average 203) were in the same range as previous RKI estimates for Germany (192 annually during 2004–2008) (*14*,*20*).

**Table 3:** Published norovirus gastroenteritis hospitalization incidence rates in selected countries in Europe and North America, 1996–2013*

Country	Data source	Study years	Infection type or diagnostic position	No. cases/10,000 population
Germany (this study)	Retrospective analysis of DESTATIS hospitalization data	2007–2012	Primary diagnosis	3.4
		2007–2012	Secondary diagnosis	4.9
Germany (*14*)	Retrospective analysis of RKI notification data	2002–2008	Nosocomial	1.6
Germany (*13*)	Retrospective analysis of RKI notification data	2001–2009	Community-acquired	1.0–2.5
England (*26*)	Retrospective regression analysis using PHE notification data and HES emergency admissions	2000–2006	Community-acquired	1.0–4.3†
		2000–2006	Community-acquired	0.23–0.48‡
England (*27*)	Retrospective analysis of GP and hospital discharge databases	2007–2013	Any position	6.2–8.0
The Netherlands (*23*)	Retrospective analysis of national surveillance data	2009	Community-acquired	1.2
United States (*24*)	Retrospective regression analysis using NIS data	1996–2007	Any position	2.1
Canada (*25*)	Retrospective binomial generalized linear analysis using CIHI-HMDB data	2006–2011	Primary diagnosis	0.05
		2006–2011	Other diagnosis	0.17

*CIHI-HMDB, Canadian Institutes of Health Information–Hospital Morbidity Database; DESTATIS, German Federal Statistics Office; HES, Hospital Episode Statistics; NIS, Nationwide Inpatient Sample; PHE, Public Health England; RKI, Robert Koch Institute. †Age >65 y. ‡Age 18–64 y.

The study years (2007–2012) preceded introduction of universal rotavirus vaccination in Germany in 2013. Considering all age groups, the number of hospitalizations caused by norovirus is markedly higher than the number caused by rotavirus. Although the highest incidence of NGE hospitalizations was in children <2 years of age, within this age group, rotavirus AGE hospitalizations continued to be more frequent.

Our study has several strengths, foremost being the data collection and recording of norovirus-related hospitalization data in Germany. We have taken advantage of the unique system in Germany, which has built-in financial incentives for norovirus testing of inpatients with AGE and mandatory reporting of laboratory-confirmed norovirus, to estimate the burden of NGE hospitalizations. This system provides a structure that, we believe, more comprehensively captures the role of norovirus in nonspecific AGE diagnoses than is possible in many other national databases. Our study used national hospitalization data, which provided age-specific information on length of hospital stay and medical costs. We used data from a 6-year study period, which enabled us to estimate NGE burden across multiple norovirus seasons.

We could not distinguish between community-acquired and nosocomial norovirus infections. An earlier study in Germany estimated 49% of all hospitalized NGE cases to be nosocomial in origin (*14*), whereas a study in Denmark put this estimate at 63% (*28*). We found that hospitalization with NGE as a secondary diagnosis represented 59% of all hospitalizations with NGE in our study, suggesting that these cases most likely represent nosocomial infections. The higher rate of secondary diagnoses among older adults compared with younger age groups further suggests that these cases actually represent a different type of hospitalization episode. The inverse age relationship might be related to poorer underlying health and longer hospital stays resulting in potentially greater exposure to a nosocomial infection. We could not exclude the possibility that some patients with NGE as a secondary diagnosis were given an unspecified AGE diagnosis as a primary diagnosis while awaiting the norovirus test results and should have had their infection counted as community-acquired instead of nosocomial. We therefore made an additional request to DESTATIS to provide us with the number of such cases and found that this combination occurred in <0.4% of patients who were given NGE as a secondary diagnosis. This finding, the consistency between our ratio of primary to secondary diagnoses to those reported by other investigators, and the age distribution support our assumption that secondary diagnoses reported by DESTATIS are most likely nosocomial NGE cases.

We were unable to include deaths in cases coded with NGE as a secondary diagnosis because we could not ascertain that NGE was the cause of death. The number of deaths attributable to NGE might thus have been underestimated.

Although the reliance upon the reporting of NGE cases to DESTATIS is another potential limitation of our study, the finding that 95.2% of the coded cases in a sample of patients had a documented positive norovirus result suggests this reliance is unlikely to have led to a major overestimation of the actual burden of NGE hospitalizations in Germany. Our findings in a single medical center might not be generalizable to the entire country. We do not expect, however, that testing rates would differ substantially between hospitals given the financial incentive and the strict quality controls carried out by MDK (*22*). From an economic perspective, our assessment of NGE hospitalization costs only considered the reimbursable fraction and did not take into account indirect or societal costs, which would have led to substantially higher cost estimates.

On the basis of our validation assessment of ICD-10 diagnostic codes compared with norovirus laboratory results, our estimated burden of NGE hospitalizations according to ICD-10 diagnostic coding might have underestimated the actual number of NGE hospitalizations. We had, a priori, assumed that all patients with AGE caused by norovirus are tested because of financial reimbursement incentives. However, our finding that 12.4% of patients with positive norovirus results did not have a corresponding NGE ICD-10 diagnostic code in the patient’s medical records suggests that underreporting of NGE hospitalization cases based on diagnostic codes has occurred. We also observed seasonality among the cases coded as unspecified AGE (ICD codes A08.3, A08.4, A08.5, and A09); peaks in winter and early spring suggest that a proportion of these cases might also be attributable to norovirus.

During the study period, most testing for norovirus in Germany was conducted by using an ELISA test. Compared with the standard reverse transcription PCR test, the sensitivity of ELISA has been estimated to be 77% and the specificity 96% (*29*). Correcting for these estimates would reduce the proportion of AGE hospitalizations caused by norovirus slightly, from an average of 13.8% to 13.2% (*30*).

In summary, by using national hospitalization discharge and cost data, we found that NGE resulted on average in 27,910 hospitalizations with NGE as a primary diagnosis and an additional 40,278 hospitalizations with NGE as secondary diagnosis annually in Germany, a number substantially higher than previously assumed. The associated reimbursed annual medical cost for community-acquired NGE was up to €42 million annually. These data could aid in the identification of target groups for future norovirus vaccination policies and could be used to assess the effects of vaccination on the NGE hospitalization burden after implementation.

Technical Appendix Burden of norovirus gastroenteritis among hospitalized patients in Germany, 2007–2012.

## References

[1] Lopman BA, Steele D, Kirkwood CD, Parashar UD. The vast and varied global burden of norovirus: prospects for prevention and control. PLoS Med. 2016;13:e1001999. 10.1371/journal.pmed.1001999 27115709 PMC4846155

[2] Hemming M, Räsänen S, Huhti L, Paloniemi M, Salminen M, Vesikari T. Major reduction of rotavirus, but not norovirus, gastroenteritis in children seen in hospital after the introduction of RotaTeq vaccine into the National Immunization Programme in Finland. Eur J Pediatr. 2013;172:739–46. 10.1007/s00431-013-1945-3 23361964 PMC7086648

[3] Bucardo F, Reyes Y, Svensson L, Nordgren J. Predominance of norovirus and sapovirus in Nicaragua after implementation of universal rotavirus vaccination. PLoS One. 2014;9:e98201. 10.1371/journal.pone.0098201 24849288 PMC4029982

[4] Payne DC, Vinjé J, Szilagyi PG, Edwards KM, Staat MA, Weinberg GA, et al. Norovirus and medically attended gastroenteritis in U.S. children. N Engl J Med. 2013;368:1121–30. 10.1056/NEJMsa1206589 23514289 PMC4618551

[5] Koo HL, Neill FH, Estes MK, Munoz FM, Cameron A, DuPont HL, et al. Noroviruses: the most common pediatric viral enteric pathogen at a large university hospital after introduction of rotavirus vaccination. J Pediatric Infect Dis Soc. 2013;2:57–60. 10.1093/jpids/pis070 23687584 PMC3656546

[6] Teunis PF, Moe CL, Liu P, Miller SE, Lindesmith L, Baric RS, et al. Norwalk virus: how infectious is it? J Med Virol. 2008;80:1468–76. 10.1002/jmv.21237 18551613

[7] Johnston CP, Qiu H, Ticehurst JR, Dickson C, Rosenbaum P, Lawson P, et al. Outbreak management and implications of a nosocomial norovirus outbreak. Clin Infect Dis. 2007;45:534–40. 10.1086/520666 17682985

[8] Lopman BA, Reacher MH, Vipond IB, Hill D, Perry C, Halladay T, et al. Epidemiology and cost of nosocomial gastroenteritis, Avon, England, 2002-2003. Emerg Infect Dis. 2004;10:1827–34. 10.3201/eid1010.030941 15504271 PMC3323246

[9] Lynn S, Toop J, Hanger C, Millar N. Norovirus outbreaks in a hospital setting: the role of infection control. N Z Med J. 2004;117:U771. 15014560

[10] [Revised case definitions for the submission of evidence of dengue virus and norovirus and morbidity or death from dengue fever and norovirus gastroenteritis.] [in German]. Bundesgesundheitsblatt Gesundheitsforschung Gesundheitsschutz. 2011;54:246–50. 21290282 10.1007/s00103-010-1214-9

[11] Uhlig U, Kostev K, Schuster V, Koletzko S, Uhlig HH. Impact of rotavirus vaccination in Germany: rotavirus surveillance, hospitalization, side effects and comparison of vaccines. Pediatr Infect Dis J. 2014;33:e299–304. 10.1097/INF.0000000000000441 24911897

[12] Kowalzik F, Zepp F, Hoffmann I, Binder H, Lautz D, van Ewijk R, et al. Disease burden of rotavirus gastroenteritis in children residing in Germany: a retrospective, hospital-based surveillance. Pediatr Infect Dis J. 2016;35:97–103. 26421806 10.1097/INF.0000000000000939

[13] Bernard H, Höhne M, Niendorf S, Altmann D, Stark K. Epidemiology of norovirus gastroenteritis in Germany 2001-2009: eight seasons of routine surveillance. Epidemiol Infect. 2014;142:63–74. 23517686 10.1017/S0950268813000435 PMC9152553

[14] Spackova M, Altmann D, Eckmanns T, Koch J, Krause G. High level of gastrointestinal nosocomial infections in the german surveillance system, 2002-2008. Infect Control Hosp Epidemiol. 2010;31:1273–8. 10.1086/657133 21047180

[15] Treanor JJ, Atmar RL, Frey SE, Gormley R, Chen WH, Ferreira J, et al. A novel intramuscular bivalent norovirus virus-like particle vaccine candidate—reactogenicity, safety, and immunogenicity in a phase 1 trial in healthy adults. J Infect Dis. 2014;210:1763–71. 10.1093/infdis/jiu337 24951828 PMC8483568

[16] Bernstein DI, Atmar RL, Lyon GM, Treanor JJ, Chen WH, Jiang X, et al. Norovirus vaccine against experimental human GII.4 virus illness: a challenge study in healthy adults. J Infect Dis. 2015;211:870–8. 10.1093/infdis/jiu497 25210140 PMC5914500

[17] Beckmann MW, Juhasz-Böss I, Denschlag D, Gaß P, Dimpfl T, Harter P, et al. Surgical methods for the treatment of uterine fibroids—risk of uterine sarcoma and problems of morcellation: position paper of the DGGG. Geburtshilfe Frauenheilkd. 2015;75:148–64. 10.1055/s-0035-1545684 25797958 PMC4361164

[18] Zwink N, Jenetzky E, Schmiedeke E, Schmidt D, Märzheuser S, Grasshoff-Derr S, et al.; CURE-Net Consortium. Assisted reproductive techniques and the risk of anorectal malformations: a German case-control study. Orphanet J Rare Dis. 2012;7:65. 10.1186/1750-1172-7-65 22978793 PMC3519554

[19] Statistisches Bundesamt Wiesbaden. GENESIS-Online [cited 2017 Dec 15]. https://www-genesis.destatis.de/genesis/online/logon?language=de&sequenz=statistiken&selectionname=12*&usg=ALkJrhjgxiAsKejwJNMswJxtZm3-LkALkA

[20] Werber D, Hille K, Frank C, Dehnert M, Altmann D, Muller-Nordhorn J, et al. Years of potential life lost for six major enteric pathogens, Germany, 2004–2008. Epidemiol Infect. 2012;•••:1–8. 22813426 10.1017/S0950268812001550 PMC9151843

[21] Hall AJ, Lopman BA, Payne DC, Patel MM, Gastañaduy PA, Vinjé J, et al. Norovirus disease in the United States. Emerg Infect Dis. 2013;19:1198–205. 10.3201/eid1908.130465 23876403 PMC3739528

[22] Medizinischer Dienst der Krankenversicherung [cited 2015 Nov 22]. http://www.mdk.de

[23] Verhoef L, Koopmans M. W VANP, Duizer E, Haagsma J, Werber D, et al. The estimated disease burden of norovirus in the Netherlands. Epidemiol Infect. 2012;•••:1–11. 22595489 10.1017/S0950268812000799 PMC9151884

[24] Lopman BA, Hall AJ, Curns AT, Parashar UD. Increasing rates of gastroenteritis hospital discharges in US adults and the contribution of norovirus, 1996-2007. Clin Infect Dis. 2011;52:466–74. 10.1093/cid/ciq163 21258098

[25] Morton VK, Thomas MK, McEwen SA. Estimated hospitalizations attributed to norovirus and rotavirus infection in Canada, 2006-2010. Epidemiol Infect. 2015;143:3528–37. 10.1017/S0950268815000734 25991407 PMC4657031

[26] Verstraeten T, Cattaert T, Harris J, Lopman B, Tam CC, Ferreira G. Estimating the burden of medically attended norovirus gastroenteritis: modeling linked primary care and hospitalization datasets. J Infect Dis. 2017;216:957–65. 10.1093/infdis/jix410 28961927 PMC5853278

[27] Haustein T, Harris JP, Pebody R, Lopman BA. Hospital admissions due to norovirus in adult and elderly patients in England. Clin Infect Dis. 2009;49:1890–2. 10.1086/648440 19911997

[28] Franck KT, Nielsen RT, Holzknecht BJ, Ersbøll AK, Fischer TK, Böttiger B. Norovirus genotypes in hospital settings: differences between nosocomial and community-acquired infections. J Infect Dis. 2015;212:881–8. 10.1093/infdis/jiv105 25701867

[29] Geginat G, Kaiser D, Schrempf S. Evaluation of third-generation ELISA and a rapid immunochromatographic assay for the detection of norovirus infection in fecal samples from inpatients of a German tertiary care hospital. Eur J Clin Microbiol Infect Dis. 2012;31:733–7. 10.1007/s10096-011-1366-z 21809086

[30] Rogan WJ, Gladen B. Estimating prevalence from the results of a screening test. Am J Epidemiol. 1978;107:71–6. 10.1093/oxfordjournals.aje.a112510 623091

