# Supplementary material for: Norovirus Gastroenteritis among Hospitalized Patients, Germany, 2007–2012
Source: Emerg Infect Dis. 2018 Nov;24(11):2021–8. doi: 10.3201/eid2411.170820 (PMC6199990; doi:10.3201/eid2411.170820)
Supplement: Technical Appendix — Burden of norovirus gastroenteritis among hospitalized patients in Germany, 2007–2012. [file 17-0820-Techapp-s1.pdf]

Article DOI: https://doi.org/10.3201/eid2411.170820

Norovirus Gastroenteritis among Hospitalized Patients, Germany, 2007–2012

Technical Appendix

Technical Appendix Table 1. Annual number and incidence rate (cases/10,000 population) of hospitalizations for norovirus gastroenteritis (NGE) (primary diagnosis) in Germany, 2007–2012*

	2007			2008			2009			2010			2011			2012		
Age group, y	N	Rate	%	N	Rate	%	N	Rate	%	N	Rate	%	N	Rate	%	N	Rate	%
<1	3,108	45.3	0.89	3,630	53.1	1.08	2,605	39.2	0.82	2,594	38.2	0.79	2,292	34.8	0.74	2,400	35.6	0.78
1	2,846	42.1	2.40	3,582	52.0	2.86	3,364	48.9	2.70	2,946	43.9	2.39	2,838	41.8	2.36	2,806	42.1	2.34
2	1,224	17.8	1.39	1,368	20.2	1.58	1,246	18.1	1.44	1,286	18.6	1.46	1,159	17.3	1.35	1,180	17.2	1.39
3	632	8.9	0.79	697	10.1	0.90	600	8.9	0.81	679	9.8	0.89	676	9.8	0.88	656	9.7	0.89
4	409	5.8	0.57	452	6.4	0.66	414	6.0	0.62	469	6.9	0.72	473	6.9	0.73	489	7.0	0.76
5	278	3.8	0.43	296	4.2	0.48	259	3.7	0.43	385	5.6	0.65	388	5.7	0.67	413	6.0	0.72
6	193	2.6	0.34	201	2.8	0.37	223	3.1	0.43	329	4.6	0.64	312	4.5	0.61	301	4.4	0.62
7	160	2.1	0.32	177	2.4	0.37	171	2.4	0.37	257	3.6	0.57	266	3.8	0.58	274	4.0	0.62
8	157	2.0	0.33	169	2.2	0.36	183	2.5	0.39	261	3.6	0.58	244	3.5	0.54	263	3.7	0.61
9	168	2.1	0.34	160	2.1	0.33	182	2.4	0.38	232	3.1	0.49	240	3.3	0.51	255	3.6	0.57
10	144	1.8	0.28	170	2.1	0.33	154	2.0	0.30	259	3.3	0.53	240	3.3	0.48	260	3.6	0.55
11	134	1.7	0.26	150	1.8	0.28	149	1.9	0.28	227	2.9	0.43	244	3.2	0.45	232	3.1	0.45
12	111	1.4	0.22	136	1.7	0.26	127	1.6	0.23	193	2.4	0.35	223	2.9	0.41	220	2.8	0.41
13	104	1.3	0.19	134	1.7	0.24	73	0.9	0.13	175	2.1	0.29	190	2.4	0.31	194	2.5	0.33
14	73	0.9	0.11	89	1.1	0.14	88	1.1	0.14	163	2.0	0.25	184	2.3	0.27	190	2.4	0.28
15	85	1.0	0.12	106	1.3	0.15	109	1.4	0.15	158	2.0	0.22	168	2.1	0.23	220	2.7	0.28
16	105	1.2	0.12	123	1.5	0.15	115	1.4	0.14	198	2.5	0.25	193	2.5	0.23	194	2.4	0.23
17	139	1.4	0.15	142	1.6	0.15	149	1.8	0.16	218	2.6	0.24	239	3.0	0.27	254	3.2	0.28
18–44	2,666	0.9	0.08	3,278	1.1	0.10	3,276	1.1	0.10	4,946	1.8	0.15	5,360	2.0	0.16	4,881	1.8	0.14
45–64	1,866	0.8	0.05	2,468	1.1	0.06	2,510	1.1	0.06	3,884	1.7	0.09	3,663	1.6	0.08	3,428	1.4	0.08
65–84	4,800	3.2	0.08	7,293	4.9	0.12	6,536	4.3	0.10	9,495	6.4	0.15	7,386	5.0	0.11	6,925	4.7	0.10
85+	2,040	11.8	0.19	3,139	17.4	0.27	2,839	15.2	0.24	4,086	20.9	0.33	3,110	16.1	0.24	3,120	15.6	0.22
All	21,442	2.6	0.13	27,960	3.4	0.17	25,372	3.1	0.15	33,440	4.1	0.20	30,088	3.7	0.18	29,155	3.6	0.17

N, number of NGE hospitalizations; Rate, incidence rate using population projections from Statistisches Bundesamt W. Statistisches Bundesamt Deutschland - GENESIS-Online. 2015; published online July 29. https://www-genesis.destatis.de/genesis/online/logon?language = de&sequenz = statistiken&selectionname = 12&usg = ALkJrhjgxiAsKejwJNMswJxtZm3-LkALkA (accessed July 29, 2015); %, NGE hospitalizations as a percentage of all hospitalizations (all cause).

Technical Appendix Table 2. Total and average number of days in hospital for norovirus gastroenteritis (NGE) (secondary diagnosis) in Germany, 2007–2012*

Age group, y	2007			2008			2009			2010			2011			2012		
	N	Days	Average	N	Days	Average	N	Days	Average	N	Days	Average	N	Days	Average	N	Days	Average
<1	1,664	28,563	17.2	1,804	27,957	15.5	1,503	23,801	15.8	1,478	26,338	17.8	1,458	30,483	20.9	1,312	22,158	16.9
1	933	7,758	8.3	1,001	8,498	8.5	943	7,760	8.2	898	6,821	7.6	881	6,819	7.7	851	5,923	7.0
2	402	3,293	8.2	355	3,169	8.9	316	2,321	7.3	337	2,597	7.7	355	2,148	6.1	344	2,520	7.3
3	204	1,581	7.8	199	1,460	7.3	168	1,839	10.9	191	1,598	8.4	160	1,193	7.5	193	1,554	8.1
4	108	958	8.9	116	969	8.4	111	863	7.8	105	856	8.2	115	655	5.7	101	670	6.6
5	92	932	10.1	70	666	9.5	65	787	12.1	87	682	7.8	78	549	7.0	105	876	8.3
6	69	729	10.6	60	785	13.1	68	1,294	19.0	60	475	7.9	68	711	10.5	54	349	6.5
7	41	388	9.5	48	334	7.0	40	326	8.2	63	505	8.0	49	440	9.0	53	362	6.8
8	49	521	10.6	52	618	11.9	45	335	7.4	56	554	9.9	48	260	5.4	44	314	7.1
9	52	539	10.4	48	441	9.2	29	279	9.6	50	535	10.7	47	405	8.6	51	360	7.1
10	52	571	11.0	37	433	11.7	45	412	9.2	55	532	9.7	55	437	7.9	45	439	9.8
11	38	519	13.7	36	314	8.7	38	246	6.5	55	859	15.6	41	525	12.8	52	333	6.4
12	41	463	11.3	32	286	8.9	43	482	11.2	43	534	12.4	46	542	11.8	62	702	11.3
13	41	352	8.6	49	762	15.6	38	451	11.9	55	677	12.3	55	665	12.1	34	493	14.5
14	35	311	8.9	42	503	12.0	31	341	11.0	44	475	10.8	42	540	12.9	48	465	9.7
15	45	641	14.2	45	819	18.2	31	311	10.0	51	402	7.9	45	426	9.5	63	919	14.6
16	50	611	12.2	37	518	14.0	38	646	17.0	37	399	10.8	39	428	11.0	44	499	11.3
17	37	311	8.4	50	800	16.0	32	277	8.7	48	423	8.8	47	443	9.4	41	450	11.0
18–44	1,285	16,429	12.8	1,355	17,069	12.6	1,314	16,901	12.9	1,992	23,918	12.0	1,639	18,379	11.2	1,494	17,855	12.0
45–64	3,865	68,660	17.8	4,588	82,532	18.0	4,462	79,141	17.7	7,181	123,283	17.2	4,598	81,483	17.7	4,345	80,928	18.6
65–84	17,325	335,785	19.4	21,609	426,169	19.7	21,108	404,582	19.2	33,133	608,734	18.4	20,167	374,039	18.5	19,743	378,765	19.2
85+	5,831	110,623	19.0	7,537	146,279	19.4	7,467	139,834	18.7	11,542	210,033	18.2	7,813	143,888	18.4	7,817	144,986	18.5
All	32,259	580,538	18.0	39,170	721,381	18.4	37,935	683,229	18.0	57,561	1,011,230	17.6	37,846	665,458	17.6	36,896	661,920	17.9

*N, number of NGE hospitalizations; Days, total number of days in hospital; Average, average days in hospital per episode.
